# Donald Trump's Name, Image, and Likeness executive order: a neo-Jacksonianism tradition

**DOI:** 10.3389/fspor.2026.1802546

**Published:** 2026-06-22

**Authors:** Billy Hawkins

**Affiliations:** Department of Health and Human Performance, University of Houston, Houston, TX, United States

**Keywords:** Name, Image, and Likeness (NIL), Andrew Jackson, college athletic reform, college athletics, Donald Trump

## Abstract

**Introduction:**

Governmental intervention in sport is not uncommon, especially at the professional level of the global sports industry. However, until recently, governmental intervention in intercollegiate sport was rare.

**Methods:**

Using a contextual analysis, this article situates Donald Trump's 2025 Name, Image, and Likeness (NIL) executive order within the historical tradition of neo-Jacksonian populism.

**Results:**

Drawing parallels to Andrew Jackson, it argues that Trump's intervention reflects exclusionary nationalism, economic populism, and executive-centered governance that expand protections for some while constraining others.

**Discussion:**

Framed as “saving college sports,” the order reasserts amateurism, restricts third-party compensation, and curtails athletes' labor rights, potentially and disproportionately affecting Black athletes in revenue-generating sports.

**Conclusion:**

The article contends that the policy preserves institutional control and racialized hierarchies, limiting economic emancipation under the guise of reform.

## Introduction

Government intervention, and more specifically presidential oversight, has taken on many forms throughout the history of the United States. For example, the concept of “Forty Acres and a Mule” is frequently misconstrued as a formal legislative statute. In actuality, it originates from *Special Field Order No. 15*, issued in January 1865 by Union General William T. Sherman during the Civil War. This military directive temporarily designated confiscated lands along the coasts of South Carolina, Georgia, and Florida for redistribution to newly liberated Black families in 40-acre parcels. The U.S. Army occasionally supplied mules as well, which led to the emergence of the phrase, “Forty acres and a Mule”. This directive facilitated the economic independence of freed Black individuals and also served to incentivize Black men to enlist in the Union forces (1).

Regrettably, this was not legislation enacted at the federal level nor passed by Congress. It constituted a military directive issued during wartime; therefore, it did not constitute federal law. Following President Lincoln's assassination, President Andrew Johnson assumed office in April 1865 and promptly revoked Sherman's order. According to various scholars, Johnson rescinded this military directive for multiple reasons. Firstly, as stated by Foner (2), Johnson's decision favored the interests of White Southern Landowners. Foner (2) asserts that,

Pardons restored not only civil and political rights but property rights, and Johnson distributed them liberally to former Confederates…In the summer of 1865, the army began evicting Black person(s)/people who had settled on “abandoned” land, restoring the property to pardoned owners (pp. 246–247).

Therefore, this pardon of ex-Confederates and the restoration of land to white planters undermined efforts to allocate property to freed or formerly enslaved Black individuals. It perpetuated the practice of sharecropping and consequently established a system of perpetual economic dependence (2).

Before Johnson demonstrated his allegiance to white supremacist ideology, where he explicitly stated that, “This is a country for white men, and by God, as long as I am President, it shall be a government for white men” [(3), p. 107], this was a practice also witnessed by one of his predecessors, Andrew Jackson. Consequently, this was a strategic political move to secure loyalty from white Southerners, used by both Jackson and Johnson, who were both slave owners. According to Trefousse (4):

Johnson's paramount goal was the speedy restoration of the Southern states to the Union, with as few changes as possible in their prewar ruling class…Restoring land to former Confederates was a central means of achieving reconciliation (p. 203).

While Jackson's commitment to white supremacist ideology informed his vehement opposition to Black suffrage and his resolve to maintain their economic and political servitude. Johnson's exercise of executive authority in this manner not only reflected his allegiance to white supremacist beliefs but also deprived the Freedman's Bureau of its power, resources, and authority to contest Johnson's reversals of land redistribution (5). Of these two former presidents, their actions reflect negatively towards certain populations, e.g., women, Black person(s)/people, and Native Americans. Both populist traditions praised the “common” man, and Trump's political philosophy has been compared to both [see e.g. (6, 7)]. However, for the focus of this article, it will compare Trump to the neo-Jacksonianism tradition.

The wielding of executive power observed during the presidencies of Andrew Jackson and Donald Trump exemplifies a notable parallel. Although the management of intercollegiate athletics has historically presented numerous challenges since its inception, the issuance of Donald Trump's executive order concerning Name, Image, and Likeness (NIL) rights in July 2025 signifies one of the most ambitious federal interventions in American college sports since Theodore Roosevelt's assembly of college presidents in 1905 to address the issue of brutality in college football. Likewise, on March 6, 2026, Trump convened more than 50 leaders from politics, media, higher education, and college and professional sports (no athletes were invited) to discuss the state of college sports. Regarding his NIL executive order, Trump's actions are presented as measures aimed at “saving college sports,” not from on-field violence but from complete disorder; the order emphasizes the principle of amateurism while restricting third-party financial arrangements that are deemed inconsistent with the educational mission of universities (8). By prohibiting donor-driven “pay-for-play” collectives, mandating that institutions safeguard women's and non-revenue sports, and instructing federal agencies to define the employment status of student-athletes, Trump's directive is more than a policy maneuver—it is a symbolic assertion of presidential authority within a domain traditionally regulated by the NCAA and state legislatures (9, 10).

This article seeks to situate Trump's NIL executive order within the broader context of neo-Jacksonianism, establishing direct parallels between Trump's governing philosophy and Andrew Jackson's populist ethos of the 19th century. Like Jackson, Trump utilizes executive authority to uphold an idealized cultural order, employs populist rhetoric to justify restrictions on economic freedom, and wields a selective form of egalitarianism that extends protections to certain groups while constraining others (11, 27). Specifically, building upon these parallels, the objective is to demonstrate how Trump's approach to college sports exemplifies the operation of neo-Jacksonian politics in the twenty-first century: concurrently populist and paternalistic, disruptive and preservative, democratic in rhetoric yet hierarchically impactful. Consequently, indirectly affecting one of the most valuable commodities within the athletic labor force—the Black male athlete.

Beyond their shared reliance on executive authority, both Jackson and Trump presided over eras defined by intense racial conflict and struggles over the boundaries of citizenship. Each used the language of “the people” to rationalize exclusionary policies that reinforced white supremacy while marginalizing non-white communities. Jackson's expansion of white male suffrage coincided with Indian removal and the entrenchment of slavery, while Trump's presidencies have been marked by systematic attacks on immigrants, Muslims, racial justice movements, and diversity, equity, and inclusion (DEI) initiatives that disproportionately target non-white populations (12, 13). Trump's public condemnation of Black athletic protest exemplifies this pattern. At a September 2017 rally in Alabama, responding to Colin Kaepernick and other NFL players kneeling during the national anthem, Trump declared: “Wouldn't you love to see one of these NFL owners, when somebody disrespects our flag, to say, ‘Get that son of a bitch off the field right now. Out! He's fired!’” [(14, 15), para 7].

This crude directive—met with roaring applause from the predominantly white crowd—illustrates how Trump's populist rhetoric intertwines with anti-Black racism and the policing of Black athletes' constitutional rights to dissent (16). His appointment of alt-right figure Steve Bannon as chief strategist and senior counselor to the president in 2016, his infamous assertion that there were “very fine people on both sides” after white supremacist violence in Charlottesville in August 2017, and his subsequent pardons of January 6 rioters all underscore his alignment with pro-white nationalist currents that parallel Jackson's white populism (17–19). Situating Trump's NIL executive order against this broader record of racialized populism clarifies how his intervention in college sports extends a longer tradition of neo-Jacksonian politics: one that expands protections for some Americans while constraining the rights and opportunities of others, particularly Black athletes in revenue-generating sports.

## Methodology and conceptual framework

This study uses a contextual analysis methodology to situate Trump's executive NIL order within a broader historical context. Contextual analysis is a qualitative methodological approach that interprets social phenomena by situating them within the historical, cultural, institutional, and political environments in which they occur. Rather than treating behaviors, texts, or events as isolated units of analysis, contextual analysis assumes that meaning is socially constructed and shaped by broader structural conditions and social processes (20, 21). Researchers employing contextual analysis examine how macro-level forces—such as economic systems, legal frameworks, organizational norms, and cultural ideologies—interact with individual experiences to produce particular outcomes. This approach often incorporates multiple forms of evidence, including policy documents, archival materials, interviews, and media sources, allowing scholars to interpret phenomena within their broader institutional and historical settings (22, 23). Contextual analysis is particularly useful for understanding complex social institutions because it connects individual experiences to larger structural dynamics.

Methodologically, contextual analysis involves an iterative interpretive process in which researchers move between empirical data and theoretical frameworks to understand how context shapes meaning and outcomes (21). Analysts identify multiple contextual layers—including temporal context (historical developments), spatial context (institutional or geographic settings), and sociopolitical context (relations of power and inequality)—and assess how these dimensions influence the phenomenon under study (20, 23). The approach emphasizes interpretive depth rather than simple description, linking specific cases to broader social processes and institutional structures. By incorporating documentary evidence and historically grounded interpretation, contextual analysis provides a systematic way to examine how social environments shape actions, policies, and experiences over time (22).

This methodology will be used to situate Donald Trump as a contemporary embodiment of Andrew Jackson; thus, it is imperative to engage with theories of populism and the concept of neo-Jacksonianism, with particular emphasis on the latter. While the term “populism” is notoriously challenging to define, scholars generally characterize it as a thin-centered ideology or political style that dichotomizes society into two opposing groups: “the pure people” and “the corrupt elite” (24). Populist leaders claim exclusive representation of the people's will, often circumventing institutions and procedural mechanisms that constrain executive authority. Within the American context, Andrew Jackson is frequently regarded as the quintessential populist president, and Donald Trump is often described as a neo-Jacksonian, reviving that style in the contemporary era (25). Both populism and neo-Jacksonianism will be employed to critically analyze President Trump's executive order concerning NIL legislation and his broader initiatives to restrict the commercialization of collegiate athletics. Consequently, akin to Jackson's anti-Black legislation and pro-white ideology, Trump's approach to college athletics reflects an evident disregard for the fundamental laborers within the collegiate athletic industrial complex.

### The concept of populism and neo-Jacksonism

Recurring waves of populist sentiment recur in American political history, moments when a political figure emerges, proclaiming to be a voice for “the people” against entrenched elites. These movements, often framed as democratizing revolts, simultaneously broaden access to political participation for some while narrowing it for others. According to Laclau (26), populism is not an ideology with a fixed content, but rather a political logic that constructs “the people” in opposition to an elite or “other”. For Jackson, the political logic of populism manifested in several ways, all of which sought to benefit white men but were at the expense of Native Americans, women, and African Americans.

For instance, Jacksonian populism was founded on the belief that political power should not be confined to the wealthy or the educated but rather extended to the “common man” (11). Consequently, the reduction of property requirements for voting, which previously restricted suffrage, facilitated greater participation among poor and working-class white men in the political process (11, 27). However, it is important to recognize that this pro-poor and working-class white men's agenda also contained elements of both expansion and exclusion: namely, the broadening of white male suffrage and the simultaneous exclusion of non-white and non-male individuals.

Both Jackson and Trump have positioned themselves as outsiders to the political establishment, cultivated personas that represent “the people,” and waged war against political elites, characteristic of populist movements. Nonetheless, neither conception of “the people” has been inclusive. As previously noted, Jackson's political philosophy and agenda were predicated upon white male suffrage. Conversely, Trump's political philosophy and agenda, particularly in its second iteration, have centered on pro-white nationalism, which has criminalized immigrants and Muslims, while *simultaneously discrediting* social justice advocates and initiatives aimed at fulfilling Civil Rights Movement principles—such as diversity, equity, and inclusion (DEI). Both agendas inherently suggest a broader democratization, purportedly expanding access to political participation for “the people”. However, in practice, these developments have resulted in a narrowing of political voice and benefits, disproportionately excluding non-white and non-male populations.

Historians and political scientists increasingly interpret Trump as a “neo-Jacksonian” figure, reviving Jackson's style of populism in a modern context (25, 28). According to these scholars, neo-Jacksonianism can be understood as a recurring strain in American political life characterized by specific features, e.g., including exclusionary policies that reinforce racial and cultural hierarchies, economic populism framed as anti-elitist but often benefiting elites, and executive-centered governance that challenges institutional checks. For brevity's sake, I will briefly address all three, but I will primarily focus on executive-centered governance in analyzing Trump's venture to save college athletics.

#### Exclusionary nationalism that reinforces racial and cultural hierarchies

As previously articulated concerning Jackson's extension of white male suffrage, his presidency exemplified the paradox inherent in American populism. On one side, Jacksonian democracy broadened political participation for ordinary white men, establishing the foundation for mass politics in the United States. Conversely, it solidified racialized hierarchies, dispossessing Native Americans, expanding slavery, and silencing women. Jackson's populism was not an all-encompassing democratization but a selective expansion benefiting some while excluding others. This affirmation of white male supremacy constituted an enduring legacy of Jacksonian exclusionary nationalism. His vision of democracy largely excluded women from formal political consideration. Thus, Jackson did not champion measures to expand women's legal or political rights, and his administration left intact a gendered legal regime that subordinated women with both the household and public spere (11, 29).

Additionally, another act that contributed to the legacy of Jacksonian democracy's exclusionary nationalism was the dispossession of Native peoples. As the architect of the Indian Removal Act (1830), he authorized the forced relocation of Native tribes from the southeastern United States to lands west of the Mississippi, a policy which Jackson justified as essential for white settlement and national development. This policy led to the Trail of Tears, resulting in the loss of thousands of Native lives and marking one of the most tragic instances of ethnic cleansing in United States history (29). Moreover, Jackson's disregard for the Supreme Court's decision in *Worcester v. Georgia* (1832) (30)—which acknowledged Cherokee sovereignty—demonstrates his contempt for judicial authority when it conflicted with his populist agenda. By prioritizing the interests of white settlers, Jackson expanded democracy for one segment of the population while denying sovereignty and survival to another.

#### Economic populism framed as anti-elitist that benefits the elite

Similar to his utilization of executive authority, Jackson's most emblematic populist campaign was against the Bank of the United States, which he depicted as a corrupt institution serving the interests of the wealthy elite at the expense of ordinary Americans. In 1832, he vetoed the recharter of the Bank, asserting that it concentrated excessive power in unelected hands and was “unauthorized by the Constitution, subversive of the rights of the States, and dangerous to the liberties of the people” [(31), para 2]. This veto was unprecedented not only in its substance but also in its employment of populist rhetoric to justify executive action. By appealing directly to the populace rather than through Congress, Jackson expanded the scope of presidential power and redefined the veto as a political instrument rather than merely a constitutional one. His victory over the Bank symbolized the triumph of the “common man” against entrenched elite interests, despite the fact that the practical outcomes more often favored state banks and speculators than ordinary farmers. Furthermore, the term “common man” was inherently myopic in scope and could be more precisely described as *“the common white man”*. Once again, Andrew's presidential perspective and focus marginalized certain segments of the population while privileging others, even in cases where he targeted entrenched elites.

#### Executive-centered governance

Perhaps the most enduring institutional legacy of Andrew Jackson was his significant expansion of presidential authority, which transformed the office into the principal embodiment of popular sovereignty. Jackson utilized the veto power more frequently than all of his predecessors combined, employing it not solely as a constitutional instrument but also as a political instrument. While earlier presidents generally limited vetoes to issues of constitutionality, Jackson used them to thwart legislation he regarded as opposing the will of the people. His 1832 veto of the recharter of the Second Bank of the United States exemplifies this methodology, as Jackson rationalized the veto by referencing democratic principles and depicting the Bank as an elitist institution that prioritized the interests of the wealthy at the expense of ordinary citizens (27, 31, 32). Consequently, this redefined the role of the presidency as a defender of the people's interests rather than merely a neutral executor of congressional legislation.

Jackson's actions stimulated considerable debate regarding executive authority. Critics leveled accusations of monarchical tendencies, referring to him as “King Andrew I” and illustrating him in political cartoons dressed in royal attire while trampling the Constitution (11). As Howe (11) further indicates, to his opponents, Jackson's defiance of Congress and the judiciary posed a threat to the separation of powers and the republican equilibrium of governance. Conversely, to his supporters, Jackson's assertive leadership embodied a necessary correction to elitist dominance. They regarded his willingness to challenge institutional norms as a demonstration of his allegiance to the common populace.

This personalization of executive authority established a precedent for subsequent populist leaders who would claim to embody the will of the people, thereby challenging institutional constraints. By positioning the president as the sole voice of the populace, Jackson helped inaugurate a tradition of plebiscitary democracy in the United States—one in which executive authority is justified not solely by constitutional limitations but also through direct appeals to popular sentiment (33). This legacy continues to resonate throughout American political history, from Abraham Lincoln's wartime expansion of executive powers to contemporary populist presidents such as Donald Trump, who portray themselves as tribunes fighting against corrupt elites on behalf of ordinary citizens. Jackson thus transformed the presidency into a more personal and populist institution, embedding ongoing tensions between democratic responsiveness and constitutional restraint that persist to this day.

This brief overview of exclusionary nationalism, executive-centered governance, and economic populism presents three exemplars that illustrate the politics of Jacksonianism. As previously noted, Jackson and Trump share analogous stylistic, ideological, and institutional characteristics. The subsequent section will demonstrate how Trump embodies neo-Jacksonianism in these domains, followed by a concluding segment that highlights executive-centered governance and its influence on collegiate sports.

## Trump's reincarnation of Jacksonianism politics

### The rise of Trump and his version of populism

Donald Trump's ascension to the presidency in 2016 marked a significant political upheaval in the United States; nonetheless, such an event was not without historical precedent. Like Jackson, who consolidated the Democratic Party as a mass political organization anchored in white male suffrage during the 1820s and 1830s, Trump rose to power as the standard-bearer of a Republican Party increasingly rooted in white working- and middle-class resentment and cultural backlash (11, 13, 28). Both leaders harnessed popular discontent to build electoral coalitions that empowered white voters while marginalizing racial minorities. Additionally, analogous to Andrew Jackson in the early nineteenth century, Trump, ironically, capitalized on popular resentment towards elites, positioning himself as the representative of the “forgotten people” or “the forgotten men and women of our country” [(34), para 1], and seeking to recalibrate political institutions to reinforce his personal authority. Cowie (34) further contends that,

Donald Trump proclaimed in his victory speech [during his first term] in the early morning hours following Tuesday's election. With his finger instinctively, almost unconsciously, on the pulse of America, he invoked a key phrase in American politics with a long and turbulent history. (para 1)

Trump also proclaimed that,

I am with you, and I will fight for you, I promise, Trump said. I will be their voice. I will be the voice for forgotten Americans in this country. (68, para 8)

Thus, his populism did not originate from a young agrarian republic but from a twenty-first-century global superpower grappling with economic dislocation, racial unrest, disdain of the voiceless, cultural change, and growing distrust in institutions. Trump's political style and policy choices exemplify how populism evolves to suit modern conditions while maintaining its core logic of expansion through exclusion.

As a master of branding and media spectacle, Trump recognized the significance of symbolic politics. From the red “Make America Great Again” hats to the orchestrated rallies, Trump transformed political support into an identity performance. Nevertheless, one of his most intentional symbolic acts was his embrace of Andrew Jackson. Shortly after assuming office during his initial presidential term, Trump displayed a portrait of Jackson in the Oval Office. He subsequently visited Jackson's tomb at The Hermitage in Tennessee, describing Jackson as a “people's president” and also stating that “he was a big fan of Jackson” [(35), paras. 1 & 4]. By paying homage and aligning himself with Jackson, Trump aimed to situate his own presidency within a revered tradition of American populism.

Positioning himself as a populist president, Trump's political rise was anchored in widespread dissatisfaction with globalization, political polarization, and declining trust in institutions. There was discontent regarding the displacement of jobs by corporate executives who aimed to increase profits and reduce labor costs by targeting labor markets advantageous to these pursuits. Some scholars contend that the 2016 election reflected a cultural backlash against increasing racial diversity, feminism, and immigration, particularly among white working- and middle-class voters who perceived themselves as displaced in a transforming America (13). Trump's slogan, “Make America Great Again,” resonated precisely because it evoked nostalgia for an imagined past when the cultural and economic dominance of white Americans was unchallenged. This nostalgic illusion and misconception enticed many to vote in favor of his presidency, not once but twice.

Trump's populism proved electorally effective. In 2016, he won the presidency while securing approximately 63 million popular votes, losing the nationwide popular vote to Hillary Clinton by nearly three million ballots, yet capturing the Electoral College (36, 37). It is interesting to note that “Andrew Jackson won by more than 10% in 1824 but was denied the presidency, which went to John Quincy Adams” [(37), para 3]. In 2024, after his 2020 defeat, he returned to office with approximately 77.3 million popular votes, representing roughly 49.9 percent of ballots cast. He defeated Kamala Harris both in the popular vote and the Electoral College, a showing that ranks among the largest raw vote totals in U.S. presidential history (38, 39). These figures underscore the depth and persistence of Trump's appeal among key constituencies, reinforcing his status as a successful neo-Jacksonian populist capable of repeatedly mobilizing a large cross-section of white voters.

Therefore, Trump's populism mirrored Jackson's white male suffrage logic by allegedly expanding political voice for a cultural majority who felt disenfranchised while suppressing others. His base was disproportionately composed of white working- and middle-class voters who felt left behind by economic globalization and cultural change (13). As stated earlier, Trump mobilized this group and proclaimed that “the forgotten men and women of our country will be forgotten no longer” [(40), para 3]. It is ironic, but simultaneously, his administration pursued policies such as voter ID laws, attacks on mail-in voting, and false claims of widespread voter fraud, all of which disproportionately disenfranchised minority voters. Similar to his hero, Trump's politics, like Jackson's, empowered one group while narrowing democracy for others, reflecting the contradiction of neo-Jacksonianism.

The paradox of expanding democracy for some while narrowing it for others becomes clearer when considered alongside contemporary demographic realities. According to the 2020 U.S. Census, non-Hispanic white Americans comprised approximately 58 percent of the population, while Black Americans accounted for about 12 percent, Latinos 19 percent, and Asian Americans roughly 6 percent (41, 42). Within this context, Trump's electoral coalition leaned heavily on white working-class voters: in 2024, as noted earlier, non-college-educated white voters supported him over Kamala Harris by approximately 66 percent to 32 percent, whereas white college graduates slightly favored Harris (41, 69). Thus, similar to Jackson's focus on white male smallholders and laborers, Trump's appeals to “the forgotten men and women of our country” effectively centered the grievances of a white majority—and particularly a white working-class bloc—while the political voice and material interests of racial minorities, both men and women, were further marginalized.

#### Trump's exclusionary nationalism that reinforces racial and cultural hierarchies

Trump's exclusionary nationalism has been evident during his first and second terms in office (Trump 1.0 and 2.0). At the heart of Trump's populism was a limited conception of who constituted “the people”. While his supporters depicted his movement as a defense of ordinary Americans, the rhetoric of Trumpism frequently characterized “the people” as white, Christian, and native-born. Trump's criticisms of immigrants, Muslims, and racial justice activists underscored how his vision of democracy was fundamentally based on exclusion.

Trump 1.0's “Muslim Ban,” officially designated as Executive Order 13769, restricted entry from several Muslim-majority countries and was justified on the grounds of national security (12). It has been regarded as the *Executive Order Protecting the Nation from Foreign Terrorists entering the United States*. Although the executive order did not explicitly specify Muslims, it alludes to an incident—the terrorists' attack on September 11, 2001—and mentions Syria, a nation known to be occupied by the Islamic State of Iraq and Syria (ISIS), a religiopolitical militant organization (43). Therefore, it can be summarized that this executive order was targeted towards Muslims.

At the southern border of the United States, Trump's initial “zero tolerance” policy resulted in the separation of families (44). The original version and implementation of this policy during Trump's first administration concluded when images depicting children being forcibly taken from their parents' arms and confined in overcrowded metal cages, resembling containment of animals, were publicly disseminated (44). Although these actions provoked widespread outrage both nationally and internationally, the subsequent policy under President Trump has expanded its scope beyond the southern border, where Immigration and Customs Enforcement (ICE) officers have been weaponized and mobilized nationwide to enforce this policy.

Domestically, Trump 1.0 persistently criticized Black Lives Matter activists and condemned NFL athletes who knelt during the national anthem, portraying their protests as unpatriotic and advocating for their dismissal. The administration of Trump 2.0 played a significant role in the removal of Black Lives Matter Plaza in Washington, D.C. (70). Additionally, H.R. 1774 was introduced by Representative Clyde Andrew of Georgia (67), stipulating that the mayor of Washington, D.C., must eliminate the plaza and rename the street Liberty Plaza, with the potential withholding of certain apportionment funds if compliance is not met. His administration, influenced by the anti-woke movement, has sought to erase Black history and other historical and cultural artifacts that reflect the diversity of this nation. Furthermore, his administration and affiliated state minions are working diligently to destroy DEI policies across several states in federally funded institutions.

The practices of Trump's 1.0 and 2.0 presidencies were and are framed as protecting the nation and restoring American greatness. However, from a critical perspective, these measures can be understood as actions that effectively reinforce racial hierarchies and cultural boundaries. Similar to Jackson's displacement of Native populations, though less violent and severe in execution, Trump's nationalism delineated the nation by excluding groups labeled as “other”. Once again, this racial and cultural hierarchy, established through these policies and practices, served to institutionalize whiteness.

#### Economic populism framed as anti-elitist that benefits the elite

This section further seeks to illustrate how Trump 1.0 and 2.0 have aligned with neo-Jacksonianism. Remember, Jackson's attack on the Bank of America represented his economic populism, thus his anti-elitist stance on behalf of the “common man”. Well, for Trump, his slogan and political ideology of “making America great again” constitutes a rebranding of a concept previously used by both former Presidents Ronald Reagan and Bill Clinton. However, Trump's employment of the term has been an effort to restore America to its glory years, when it was economically and politically strong, with a robust military to protect it from both foreign and domestic threats.

For many Americans, predominantly Republicans, Trump's political ideology resonated, thus explaining his two-term election. His popularity and support among the “common man” have been notable and ironic, given his status as a billionaire. To elaborate, prior to his first term, his net worth was estimated at $4.5 billion (45). In 2025, *Forbes* announced that the president is “now worth a record $7.3 billion, up from $4.3 billion in 2024, when he was still running for office” [(46), para 1]. His lifestyle and economic ethos stand in stark contrast to the audience he has captivated. Thus, the charisma he has deployed has enabled him to overshadow and bridge the widening gap between him and his supporters.

Furthermore, the irony of a multibillionaire leading an anti-elitist campaign is apparent in Trump 2.0's multi-faceted assault on esteemed educational institutions (such as Ivy League establishments like Harvard and Columbia), political elites (particularly Democratic leaders, irrespective of their identities), and his utilization of tariffs to dismantle the global trading system that proponents of Make America Great Again perceive as the treacherous self-enrichment of global elites [(47), para 3]. This movement has been characterized as the “anti-elite” elite (48). Genieys and Darviche (48) further observe that this “anti-elite” or the new Trumpian elite consists of prominent economic figures, including Jeff Bezos of Amazon, Mark Zuckerberg, Elon Musk, and Marc Andreessen. This eclectic group of billionaires has demonstrated, either directly or indirectly, their allegiance to Trump's ideology, thereby aligning with neo-Jacksonian principles.

To further expound, this revote of the elites or the anti-elitist's campaign is not anti-democracy:

……but the twist is that this revolt is undertaken in the name of the people. In other words, the modern idea of the people has been completely split from the idea of demos and democracy. This is a revolt (in the sense that uprisings to seize power are always revolts), but not a revolution, intended to change something in the fundamental order of polity or economy. This revolt is the effort by one elite to replace another. [(49), para 8]

Therefore, replacing one group of elites with another is a conclusion and the basis of Trump's economic populism. According to Appadurai (49), this transfer of power from one elite group to another will have some trickle-down benefits. However,

The trickledown of the more quotidian benefits, such as jobs, health care, higher incomes, and safer cities, still calls for endless patience from those at the bottom of the pyramid. If hate can trickle down to them, so perhaps can prosperity. [(49), para 14]

Finally, the “No Kings” movement, initiated on October 18, 2025, has been antithetical to this transfer of power (50). This movement disputes the idea of Trump's anti-elitist campaign, which is purportedly in the name of the people, with the emphatic assertion that “America has no kings and the power belongs to the people” [(50), para 2].

At this point, this overview endeavors to highlight Trump's resurgence of Jacksonian principles. The concluding section will briefly analyze Trump's executive-centered governance to further illustrate how his political ideology aligns with neo-Jacksonianism. Furthermore, it will serve as the central focus of the discussion concerning Trump's initiatives to safeguard collegiate athletics, which constitutes the primary objective of this article.

#### Executive-centered governance

The assertion that the leader exclusively represents the populace, thereby rendering institutional checks ineffective when they oppose the leader's will, constitutes a fundamental peril of neo-Jacksonian populism. Trump, akin to Jackson, transformed the presidency into a profoundly personal institution. His reliance on Twitter enabled him to communicate directly with millions of supporters, circumventing both traditional media and government institutions (51). His rallies, reminiscent of Jackson's stump speeches, became ritualized displays of loyalty and grievance. By demanding personal allegiance and framing himself as the singular voice of the people, Trump blurred the distinction between leader and institution.

This personalization of power, while charismatic to his base, has eroded the institutional norms of democracy. As opponents of Jackson anticipated, Jackson had become akin to “King Andrew”. Critics accused Trump of exhibiting authoritarian tendencies, which, as previously mentioned, served as a catalyst for the “No Kings” protests organized nationwide. His authoritarian inclinations threaten the system of checks and balances. In both instances, populist personalization has redefined the presidency in a manner that prioritizes loyalty to the leader over fidelity to the institution. This phenomenon is recognized within his inner circle (52) and is evidenced by his ability to exercise and expand his executive authority through various executive orders (53). Some members of his loyalist inner circle have maintained their allegiance from Trump 1.0 through his legal challenges in Manhattan to the inauguration of Trump 2.0.

Finally, Trump 2.0 has issued 217 executive orders within the initial months of his presidency. While the modern numbering system for executive orders did not exist in Jackson's era, historical estimates indicate that Jackson issued approximately twelve formal executive orders alongside numerous proclamations and unilateral actions, including his famous veto of the Bank's recharter and his defiance of Supreme Court authority in *Worcester v. Georgia* (30, 54). The contrast between Jackson's relatively small number of formally recorded orders and Trump's prolific use of executive directives underscores how neo-Jacksonian executive-centered governance has intensified in the contemporary administrative state. In this context, presidents can reshape policy across multiple domains—including college sports—through a high volume of unilateral actions (53).

The remainder of this article will explore Trump's assertion of leadership through his employment of executive orders. This executive-centric governance shall be used specifically to examine Trump's efforts to preserve college athletics. Mirroring Jackson's efforts to protect various causes via his executive-centric leadership, as previously mentioned (e.g., the common man, the federal union, etc.), Trump's Executive Order 14322, signed on July 24, 2025, represents his attempt to exert his presidential authority over college athletics.

### Saving college athletics executive order

The policy trifecta comprising NIL, the transfer portal, and revenue sharing has precipitated significant upheavals within intercollegiate athletics and has challenged the traditional paradigm of amateurism. NIL has granted athletes the opportunity to monetize their name, image, and likeness. Historically, rights to athletes' NIL were considered to be “owned” by their respective institutions. The transfer portal has facilitated a form of collegiate athlete free agency, enabling athletes to transfer without the obligation to sit out a year. To date, the Power Five Conferences (SEC, ACC, Big Ten, and Big 12) have adopted the revenue-sharing initiative. Beginning in 2025, this policy mandates these institutions to distribute approximately $20.5 million in revenue to athletes across various sports. This trifecta, arising from diverse levels of legal activism [e.g., Keller & Hart v. NCAA, (76); O'Bannon v. NCAA, (77); NCAA v. Alston, (55); House v. NCAA, (78)], has fundamentally transformed the landscape of college athletics, particularly at the FBS level.

The NCAA's former amateurism model faced significant challenges and ultimately capitulated to state-level NIL legislation, culminating in the NCAA's adoption of an interim NIL policy in 2021 (56), followed by a comprehensive policy in 2024 (57). Despite the NCAA's efforts to establish regulations aimed at containing or managing the emerging momentum—particularly in revenue-generating sports—it has lost its grasp and institutional stability amidst the profound transformations caused by NIL legislation, the transfer portal, and revenue sharing.

Notably, many of the most consequential shifts away from the NCAA's traditional amateurism model occurred during President Joe Biden's administration, prior to Trump's NIL executive order. In June 2021, under mounting pressure from state NIL laws and the Supreme Court's unanimous decision in NCAA v. Alston (55), the NCAA adopted an interim NIL policy that allowed athletes to profit from endorsements while maintaining their eligibility (56). In 2024, the association implemented expanded NIL, health, and academic benefits for Division I athletes, institutionalizing forms of compensation and support that further eroded the old amateurism paradigm (57). The subsequent *House v. NCAA* settlement in 2025 opened the door for direct revenue sharing at opt-in Division I schools, deepening the sense among many stakeholders—particularly those invested in preserving institutional control—that the system was descending into chaos (71, 72). These developments, all unfolding under a Democratic administration, primed the political space for a populist leader like Trump to claim the mantle of “saving” college sports by reasserting federal authority and rolling back athlete empowerment.

Furthermore, it is interesting to note that, in the aftermath of this policy trifecta, what some have called the wild, wild west has emerged. Magnusen & Todd (73) suggest NIL not only mimics the Wild West, but also the Dot.com era. They suggest that

…. invoking the “Wild West” is typically used to reference a chaotic environment that is desperately in need of policing via an authority figure such as a sheriff or marshal ((73), p. 14).

Furthermore, regarding the lack of guidance on NIL legislation, former Penn State football coach James Franklin describes the new NIL era as “a little bit of the wild, wild west” [(58), para 8].

An effort to regulate this wild, wild west came in July 2025 when Donald Trump issued an executive order concerning NIL rights. This executive order stands as one of the most significant governmental interventions in collegiate athletics in United States history since Theodore Roosevelt's era, as previously noted. It signifies a concerted effort to uphold the amateur status of collegiate sports by resisting what the administration perceives as the encroachment of professionalism through third-party NIL payments. Furthermore, it reaffirms the traditional perspective that college athletes are primarily students—not paid professionals—thereby limiting their capacity to monetize athletic participation beyond fair-market-value endorsements (8).

Thus, marketed as a measure to “save college sports” from chaos, the order reasserts the principle of amateurism while curbing third-party financial arrangements deemed inconsistent with the educational mission of universities (8). By banning donor-driven “pay-for-play” collectives, requiring institutions to protect women's and non-revenue sports, and directing federal agencies to clarify student-athlete employment status, Trump's directive is not merely a policy maneuver—it is a symbolic assertion of presidential authority into a domain historically governed by the NCAA and state legislatures (9, 10).

At the level of political economy, the emphasis on preserving amateurism and banning so-called pay-for-play collectives operates as a mechanism to stabilize what Hawkins (59) terms the “athletic industrial complex,” a system in which predominantly white-owned institutions, media corporations, and athletic apparel companies capture the overwhelming share of revenues generated by a disproportionately Black labor force in football and men's basketball. Power Five football and men's basketball programs, which account for the lion's share of NCAA revenues, are staffed heavily by Black athletes, even as the broader student bodies, governing boards, and administrative leadership of these universities remain majority white (59, 74).

Critics of athlete empowerment reforms frequently frame revenue-sharing and robust NIL rights as rewards for “undeserving” Black “kids” who, in their view, should be satisfied with scholarships and the symbolic prestige of representing the university—a discourse that racializes notions of merit and entitlement in ways that mirror earlier defenses of racialized labor exploitation (59). Connecting the largely white working- and middle-class critics of college athlete empowerment to Trump's populist base further illuminates the political logic at work: Trump's defense of amateurism and “traditional” college sports helps protect white institutional control and capital accumulation while constraining the economic rights of athletes, especially Black men in revenue-generating sports. This dynamic extends what Hawkins (59) has described as “The New Plantation,” a racialized division of labor in which white administrators, coaches, donors, and media partners extract disproportionate value from predominantly Black athletic labor—into the NIL era, preserving the pre-existing racial and economic status quo in ways analogous to Jackson's racist oppression and desire to keep Black person(s)/people enslaved.

As previously articulated, this executive order, entitled “Saving College Sports,” was issued amidst increasing controversies pertaining to NIL collectives, booster payments, and inconsistent state-level NIL legislation, which are fragmenting the college athletics domain. Drawing upon multiple sources, the following delineates the five principal provisions of the executive order.

1. **Ban on Third-Party Pay-for-Play Arrangements—**The provision bans booster- or donor-driven third-party NIL arrangements used as recruiting inducements—what the administration termed pay-for-play deals—while explicitly permitting fair-market-value compensation for legitimate services such as brand endorsements (10, 60).
2. **Mandated Protection of Non-Revenue and Women's Sports**—This provision will establish federal requirements to maintain or increase support for sports that do not generate significant revenue. For example, institutions with athletics revenue above $125 million must expand opportunities for women's and Olympic sports, thereby increasing scholarships and roster spots for non-revenue sports. Those with $50 million–$125 million must at least maintain their existing levels. Finally, institutions with $50 million or less must avoid disproportionate cuts to these programs [(61), para 12–15; Trump's Executive Order, 2025, para 7–9].
3. **Clarification of Student-Athlete Employment Status**—This provision of the order directs the Department of Labor and the National Labor Relations Board to issue guidance clarifying whether collegiate athletes should be considered employees. The goal is to prevent athlete unionization and reinforce the notion that college sports should remain an educational pursuit, thereby reducing their professionalization (9, 10).
4. **Federal Agency Coordination and Legal Defense**—This provision tasked multiple federal agencies to develop strategies to defend Title IX compliance and litigate against destabilizing lawsuits. These agencies include the Departments of Education, Justice, Labor, Health and Human Services, and the Federal Trade Commission. They are instructed to develop regulatory, enforcement, and litigation strategies (within 30–60 days) to preserve scholarships, defend Title IX protections, and guard against destabilizing lawsuits or state-level irregularities (61).
5. **Preservation of the Collegiate-to-Olympic Pipeline**—Finally, this executive order emphasizes that U.S. collegiate athletics are instrumental in preparing athletes for the Olympic and Paralympic teams, recognizing that a significant portion of Team USA athletes come from college sports. Therefore, amateurism, which undergirds these non-revenue-generating sports, is cast as a patriotic duty (10).

The impetus for this executive order and the provisions mentioned above is best captured in the White House statement:

President Trump recognizes the critical role of college sports in fostering leadership, education, and community pride, the need to address urgent threats to its future, including endless litigation seeking to eliminate the basic rules of college sports, escalating private-donor pay-for-play payments in football and basketball that divert resources from other sports and reduce competitive balance, and the commonsense reality that college sports are different than professional sports. [(8), para 8]

Therefore, saving college sports is saving a viable American institution. An institution with hundreds of years of rich educational, cultural, and athletic traditions. The path of college athletics has vacillated since its inception; however, not to this magnitude, especially with not so much at stake. But does college athletics require government intervention from the highest office? Does it require an executive order of this nature? How does this mimic a neo-Jacksonian philosophy?

As noted earlier, Jackson positioned himself as a defender of the “common man” or ordinary farmers, protecting them against the wealthy banking elite. As a savior of college athletics, Trump is positioning himself as a protector of athletes, especially for those who participate in non-revenue-generating sports. With his executive order, he is protecting college athletes from wealthy boosters, predatory agents, and the incoherent state NIL laws (10). The parallel between Jackson and Trump is that their protection was duplicitous and not really for the common man or the college athlete, but for institutional order. As Remini (27) noted, Jackson destroyed the Bank ostensibly to empower the common man, but in reality, he preserved the dominance of state banks and landed elites. Trump's alleged protection seeks to restrict NIL and preserve the ideals of amateurism, which have been pervasive in maintaining institutional control and economic viability (60).

Furthermore, both cases of protection required the employment of executive supremacy. With Jackson, he exercised veto power to an unprecedented degree and resisted institutional checks on his power in his effort to defend the common man (75). Trump is using an executive order to impose sweeping changes to protect college athletes, despite congressional gridlock over NIL legislation. Thus, Trump's action is a neo-Jacksonian logic of executive supremacy, where he asserts himself as the direct protector of the people against corrupt or ineffectual intermediaries (62).

Furthermore, for both leaders, executive orders exemplify a form of selective egalitarianism. According to Howe (11), Jackson expanded suffrage for white men while dispossessing Native peoples of their land and maintaining the institution of slavery. Although not to the same degree of severity, Trump has endeavored to expand protections for Olympic and women's sports (61) while simultaneously restricting the economic emancipation of many Black male athletes, as well as other athletes involved in revenue-generating sports. It is important to note that Trump's provision #2 invokes the protection of women's and Olympic sports as a central justification—mandating that institutions maintain or expand opportunities for these programs—this limited, sport-specific protection for (some) women athletes stands in notable contrast to Jackson's era, which offered no analogous executive efforts to promote women's opportunities in public life. This contrast underscores how neo-Jacksonianism can incorporate selectively gender-inclusive rhetoric to legitimize policy interventions while leaving broader hierarchies of power and privilege intact (11).

Therefore, this protection of women's sport is not pro-women sports in practice as much as it is anti-Black racism. It is well documented that Black male athletes comprise a substantial portion of the athletic labor force in basketball and football [see, e.g., (59, 74)]. According to On3, six of the top ten athletes with the highest NIL value are Black male athletes (63). Consequently, this executive order has a detrimental impact on Black male athletes in revenue-generating sports, specifically football and basketball. Therefore, once again, the desire to keep Black person(s)/people exploitable is evident while the opportunity for Black wealth accumulation is being challenged. On a smaller, yet significant scale, this mirrors how institutionalized whiteness, through governmental intervention, employs legislation that adversely and disproportionately impacts Black individuals. It is important to clarify that this governmental intervention does not solely impact Black athletes; however, there exists a historical pattern whereby legislation has been an instrument of white supremacy, weaponized to hinder Black economic emancipation.

## Conclusion

This article's central premise endeavors to contextualize Trump's NIL executive order within the broader historical tradition of neo-Jacksonianism. It draws comparisons between Trump's governing philosophy and the populist ethos of Andrew Jackson in the nineteenth century. Similar to Jackson, Trump utilizes executive authority to uphold an idealized cultural order, employs populist rhetoric to justify restrictions on economic freedom, and exhibits a selective form of egalitarianism that offers protections to certain groups while limiting others (11) (27). Trump's NIL executive order exemplifies the operation of neo-Jacksonian politics in the twenty-first century: simultaneously populist and paternalistic, disruptive and preservative, and rhetorically democratic yet hierarchically structured in its impact. Its legislative measures and regulatory control exemplify the persistence of institutionalized whiteness, whereby oversight and implementation remain predominantly under the leadership of white males.

Jackson's political ideology was driven by a longing for a virtuous agrarian past (27). Similarly, Trump's NIL policy evokes nostalgia. He venerates amateur athletics as a distinctively American institution associated with education, patriotism, and Olympic preparation (10). Both leaders invoke a mythologized past—whether it be the yeoman republic or the amateur ideal—to justify restrictive policies enacted during their respective presidencies.

Trump's NIL executive order transcends mere sports policy; it serves as a contemporary manifestation of neo-Jacksonian populism that wields executive authority to preserve racialized hierarchies under the guise of protecting tradition and fairness. By reinforcing amateurism in a context where Black athletes constitute the majority of the labor force in flagship football and men's basketball programs, the order effectively preserves a racialized division of labor in which white administrators, coaches, donors, and media partners extract disproportionate value from predominantly Black athletic labor—a dynamic that Hawkins (59) conceptualizes as “the new plantation” within the broader athletic industrial complex. Much like Andrew Jackson's oppressive racist practices and disenfranchisement of minorities (64), Trump's intervention ostensibly offers protection to certain groups (women's sports, non-revenue programs) while constraining the economic emancipation of Black male athletes who generate the majority of college sports revenue. In both instances, the narrative of a savior—whether preserving democracy in Jackson's era or safeguarding college sports in Trump's—obscures a more profound reality: selective empowerment coupled with systemic exclusion.

Consequently, although the order is ostensibly designed to protect college athletes and ensure the future viability of college sports, it effectively constrains athletes' economic independence—particularly those involved in revenue-generating sports such as football and men's basketball, where Black athletes constitute the majority of the athletic workforce, as previously indicated. Trump's intervention to preserve college sports exemplifies how neo-Jacksonian politics continues to operate in the twenty-first century, transforming college athletics into a contentious arena where issues of race, economics, tradition, and executive authority intersect. This contextual analysis elucidates how political power at the national level can be evoked to impact power dynamics at the institutional level. In this case, it is an effort to further institutionalize whiteness and minimize Black empowerment.

In conclusion, two emerging developments for future research include the oversight by the College Sports Commission (CSC) and the potential passage of the SCORE Act. Although the CSC does not have a direct connection to Trump's NIL executive order, it aligns with certain principles of the order by regulating NIL agreements, prohibiting third-party pay-for-play payments, and aiming to safeguard non-revenue sports, particularly women's sports. The CSC was established by the Power 4 conferences to oversee NIL transactions exceeding $600.00 (65). Consequently, this governing entity will function as an enforcement mechanism, either directly or indirectly, guided by the framework established by President Trump's executive order. Its relevance and effectiveness merit further scholarly inquiry.

The Student Collegiate Opportunities, Resources, and Education (SCORE) Act presents an additional avenue for further scholarly inquiry, should it garner support in Congress and be enacted as a federal NIL framework (66). This prospective legislation also corresponds with the principles outlined in Trump's executive order. Its primary objective will be to establish coherence amidst the diverse array of state-level NIL statutes. Most notably, the bill aims to “preempt state laws with respect to compensation, payments, benefits, employment status, eligibility, and academic standards applicable to student athletes” [(66), para 6]. The enactment of this legislation could potentially induce a significant transformation within the current landscape of athlete compensation.

Finally, intercollegiate athletics have not experienced such a level of governmental intervention and scrutiny from the highest office since President Theodore Roosevelt's 1905 intervention aimed at reducing violence in collegiate football. The implications of this development and the precedents it establishes merit further analysis. Moreover, the petitions for congressional oversight, the establishment of the Collegiate Sports Council (CSC), and the potential enactment of the SCORE Act will sustain the contentious nature of college athletics. Will this governmental intervention enhance educational outcomes for college athletes while preserving a financially viable commercial enterprise? Alternatively, analogous to Andrew Jackson's repressive and racist practices, which sought to keep Black person(s)/people enslaved and exploitable, will this governmental intervention diminish the prospect of economic emancipation for numerous college athletes, who have been deprived of sharing in the economic gains and profits derived from their athletic labor?

## Data Availability

The original contributions presented in the study are included in the article/Supplementary Material, further inquiries can be directed to the corresponding author.

## References

[1] Myers B. Sherman's Field Order No. 15. *New Georgia Encyclopedia*. University of Georgia Press (2020). Available online at: https://www.georgiaencyclopedia.org/articles/history-archaeology/shermans-field-order-no-15 (Accessed March 9, 2025).

[2] Foner E. Reconstruction: America's Unfinished Revolution, 1863–1877*.* New York: Harper & Row (1988).

[3] Perman M. Emancipation and Reconstruction, 1862–1879. 2nd ed. New York: W. H. Norton (2003).

[4] Trefousse HL. Andrew Johnson: A biography. New York: W. W. Norton & Company (1989).

[5] Du Bois WEB. Black Reconstruction in America, 1860–1880*.* New York: Free Press (1998). (Original work published 1935).

[6] Milkis S Tichenor D. Why Donald Trump is more dangerous than Andrew Jackson. *The Washington Post* (2019). Available online at: https://www.washingtonpost.com/outlook/2019/10/01/why-donald-trump-is-much-more-dangerous-than-andrew-johnson/ (Accessed August 10, 2025).

[7] Hamilton DS. Trump's Jacksonian foreign policy and its implications for European security. *UILbrief, No. 2* (2017). Available online at: https://www.ui.se/globalassets/butiken/ui-brief/2017/hamilton-ui–brief.-05-23.pdf (Accessed August 10, 2025).

[8] White House. Fact sheet: President Donald J. Trump saves college sports. *The White House* (2025). Available online at: https://www.whitehouse.gov/fact-sheets/2025/07/fact-sheet-president-donald-j-trump-saves-college-sports (Accessed August 10, 2025).

[9] Weissert W. Trump signs order to clarify college athletes' employment status amid NIL chaos. *AP News* (2025). Available online at: https://apnews.com/article/fc81ce9ab3813e02b763494223930a93 (Accessed August 10, 2025).

[10] Graham BA. Trump signs executive order to rein in “chaotic” influence of money on college sports. *The Guardian* (2025). Available online at: https://www.theguardian.com/sport/2025/jul/24/trump-college-sports-executive-order-nil (Accessed August 10, 2025).

[11] Howe DW. What Hath God Wrought: The Transformation of America, 1815–1848. New York: Oxford University Press (2007).

[12] Executive Order Protecting the Nation from Foreign Terrorist Entry into the United States. *Executive Orders* (2017). Available online at: https://trumpwhitehouse.archives.gov/presidential-actions/executive-order-protecting-nation-foreign-terrorist-entry-united-states/ (Accessed August 10, 2025).

[13] Norris P Inglehart R. Cultural Backlash: Trump, Brexit, and Authoritarian Populism*.* Cambridge: Cambridge University Press (2019).

[14] BBC News. Trump: NFL kneelers ‘maybe shouldn't be in country’ (2018). Available online at: https://www.bbc.com/news/world-us-canada-44232979 (Accessed August 27, 2025).

[15] Colvin J Lucey C. Trump on NFL anthem protests: ‘Get that son of a bitch off the field.’ *Times of Israel* (2017). Available online at: https://www.timesofisrael.com/trump-on-nfl-anthem-protests-get-that-son-of-a-bitch-off-the-field/ (Accessed August 27, 2025).

[16] Serwer A. Trump's war of words with Black athletes. *The Atlantic* (2017). Available online at: https://www.theatlantic.com/politics/archive/2017/09/trump-urges-nfl-owners-to-fire-players-who-protest/540897/ (Accessed August 27, 2025).

[17] Dwyer C. Trump taps Reince Priebus as chief of staff, Steve Bannon as chief strategist. *NPR* (2016). Available online at: https://www.npr.org/sections/thetwo-way/2016/11/13/501937200/trump-taps-reince-priebus-as-chief-of-staff-steve-bannon-as-chief-strategist (Accessed August 27, 2025).

[18] Holan AD. In context: Trump's ‘very fine people on both sides’ remarks. *PolitiFact* (2019). Available online at: https://www.politifact.com/article/2019/apr/26/context-trumps-very-fine-people-both-sides-remarks/ (Accessed August 27, 2025).

[19] Dunn A. Fact check: Meme on Trump ‘very fine people’ quote has inaccuracies*. *USA Today* (2020). Available online at: https://www.usatoday.com/story/news/factcheck/2020/10/17/fact-check-trump-quote-very-fine-people-charlottesville/5943239002/ (Accessed August 27, 2025).

[20] Creswell JW Poth CN. Qualitative Inquiry and Research Design: Choosing Among Five Approaches. 4th ed. Los Angeles: Sage (2018).

[21] Maxwell JA. Qualitative Research Design: An Interactive Approach. 3rd ed. Los Angeles: Sage (2013).

[22] Bowen GA. Document analysis as a qualitative research method. Qual Res J. (2009) 9(2):27–40. 10.3316/QRJ0902027

[23] Yin RK. Case Study Research and Applications: Design and Methods. 6th ed. Los Angeles: Sage (2018).

[24] Mudde C Rovira Kaltwasser C. Populism: A Very Short introduction*.* New York: Oxford University Press (2017).

[25] Mead WR. The Jacksonian revolt: American populism and the liberal order. Foreign Aff. (2017) 96(2): 2–7.

[26] Laclau E. On Populist Reason*.* New York: Verso (2005).

[27] Remini RV. Andrew Jackson and the Course of American Democracy, 1833–1845. New York: Harper & Row (1984).

[28] Meacham J. The Soul of America: The Battle for our Better Angels*.* New York: Random House (2018).

[29] Saunt C. Unworthy Republic: The Dispossession of Native Americans and the Road to Indian Territory. New York: W. W. Norton & Company (2020).

[30] Garrison TA. Worcester v. Georgia, 1832. *New Georgia Encyclopedia* (2004). Available online at: https://www.georgiaencyclopedia.org/articles/government-politics/worcester-v-georgia-1832/#:∼:text=In%20Worcester%20v.,several%20treaties%20with%20the%20Cherokees (Accessed September 3, 2025).

[31] Jackson A. President Jackson's Veto Message Regarding the Bank of the United States. Avalon Project, *Yale Law School* (1832). Available online at: https://avalon.law.yale.edu/19th_century/ajveto01.asp (Accessed September 3, 2025).

[32] Jackson A. “The veto message regarding the bank of the United States, July 10, 1832”. In: Kaminski JP, editor. The Documentary History of the Supreme Court of the United States, 1789–1800. Madison: University of Wisconsin Press (1987). p. 233–40. Original work published 1832.

[33] Skowronek S. Presidential Leadership in Political Time: Reprise and Reappraisal. Lawrence: University Press of Kansas (2006).

[34] Cowie J. Donald Trump and History's Competing Visions of America's ‘Forgotten Man.’ *TIME* (2016) Available online at: https://time.com/4567949/forgotten-man-donald-trump/ (Accessed October 8, 2025).

[35] Myer H Ebert J. Trump tours The Hermitage in Nashville, visits Andrew Jackson's tomb (2017). Available online at: https://www.usatoday.com/story/news/nation-now/2017/03/15/trump-tours-hermitage-nashville-visits-andrew-jacksons-tomb/99234880/ (Accessed October 8, 2025).

[36] 270toWin. Presidential election of 2016 (2016). Available online at: https://www.270towin.com/2016-election/ (Accessed October 8, 2025).

[37] Krieg G. It's official: Clinton swamps Trump in popular vote. *CNN* (2016). Available online at: https://www.cnn.com/2016/12/21/politics/donald-trump-hillary-clinton-popular-vote-final-count (Accessed October 8, 2025).

[38] Lindsay JM. The 2024 election by the numbers. *Council on Foreign Relations* (2024). Available online at: https://www.cfr.org/articles/2024-election-numbers (Accessed October 8, 2025).

[39] Ziegler M. Popular vote: how Trump's 77.3 million ranks among past elections. *LiveNow FOX* (2025). Available online at: https://www.livenowfox.com/news/presidents-most-popular-votes-trump-2024 (Accessed October 8, 2025).

[40] Jackson D. Stanglin D. Trump is now president: ‘The forgotten … will be forgotten no longer.’ *USA Today* (2017). Available online at: https://www.usatoday.com/story/news/politics/2017/01/20/donald-trump-inauguration-day-president-white-house/96782700/#:∼:text=In%20a%20frequently%20dark%20address,right%20now%2C%22%20he%20said.&text=Wearing%20an%20overcoat%2C%20bright%20red,%2C%20many%20years%20to%20come.%22&text=He%20pledged%20to%20transfer%20power,to%20eradicate%20%22radical%20Islam.%22 (Accessed October 8, 2025).

[41] Dilulio JJ Jr. The 4 working-class votes. *Brookings Institution* (2024). Available online at: https://www.brookings.edu/articles/the-4-working-class-votes (Accessed October 8, 2025).

[42] Jensen E. Measuring racial and ethnic diversity for the 2020 Census. *U.S. Census Bureau* (2021). Available online at: https://www.census.gov/newsroom/blogs/random-samplings/2021/08/measuring-racial-ethnic-diversity-2020-census.html (Accessed October 8, 2025).

[43] Hassan H. The secret world of Isis training camps—ruled by sacred texts and the sword. *The Guardian* (2015). Available online at: https://www.theguardian.com/world/2015/jan/25/inside-isis-training-camps (Accessed October 8, 2025).

[44] Singh M. Trump revives family separations amid drive to deport millions: ‘A tactic to punish.’ *The Guardian* (2025). Available online at: https://www.theguardian.com/us-news/ng-interactive/2025/oct/02/trump-immigration-family-separations-deportations (Accessed October 8, 2025).

[45] Fontevecchia A. Forbes' 2016 presidential candidate wealth list. *Forbes* (2015). Available online at: http://search.proquest.com.ezproxy.lib.uh.edu/magazines/forbes-2016-presidential-candidate-wealth-list/docview/1725046304/se-2 (Accessed October 8, 2025).

[46] Alexander D. Presidency boosts Trump's net worth by $3 billion in a year. *Forbes* (2025). Available online at: https://www.forbes.com/sites/danalexander/2025/09/09/presidency-boosts-trumps-net-worth-by-3-billion-in-a-year/ (Accessed October 8, 2025).

[47] Collinson S. Trump's assault on elites encompasses almost every aspect of American life. *CNN* (2025). Available online at: https://www.cnn.com/2025/03/24/politics/trump-bondi-elite-universities-boasberg-judges-deportation (Accessed October 8, 2025).

[48] Genieys W Darviche MS. Trump 2.0: the rise of an ‘anti-elite’ elite in US politics. Conference Sciences Po (2025). Available online at: https://conference.sciencespo.fr/content/2025-01-29/trump-2-0-the-rise-of-an-anti-elite-elite-in-us-politics_Jbwsfh708ZfcRL6FgSEw (Accessed October 8, 2025).

[49] Appadurai A. The revolt of the elites: when ‘populist elites from above' revolt against democracy. *Albert Hirschman Centre on Democracy* (2020). Available online at: https://www.graduateinstitute.ch/communications/news/revolt-elites (Accessed October 8, 2025).

[50] No Kings. No thrones. No crowns. No kings (2025). Available online at: https://www.nokings.org (Accessed October 8, 2025).

[51] Hacker JS Pierson P. Let Them Eat Tweets: How the Right Rules in an Age of Extreme Inequality*.* New York: Liveright Publishing Corporation (2020).

[52] Burns D Messerly M. The twenty-one people in Trump world you need to know (2025). Available online at: https://www.politico.com/news/2025/01/21/trump-world-21-people-to-know-00198691 (Accessed August 27, 2025).

[53] Federal Register. 2025 Donald J. Trump Executive Orders. *National Archives* (2025). Available online at: https://www.federalregister.gov/presidential-documents/executive-orders/donald-trump/2025 (Accessed January 3, 2026).

[54] Crews CW. “Presidential executive orders and executive memoranda”. In: Ten Thousand Commandments 2017. Competitive Enterprise Institute (2017). p. 23–6. Available online at: https://cei.org/wp-content/uploads/2017/05/Chapter_3_Ten_Thousand_Commandments_2017.pdf (Accessed January 3, 2026).

[55] *NCAA v. Alston* (2021). Available online at: https://www.supremecourt.gov/opinions/20pdf/20-512_gfbh.pdf (Accessed January 3, 2026).

[56] Hosick MB. NCAA adopts interim name, image, and likeness policy. *NCAA* (2021). Available online at: https://www.ncaa.org/news/2021/6/30/ncaa-adopts-interim-name-image-and-likeness-policy.aspx (Accessed January 3, 2026).

[57] Wright MD. New NIL, health and academic benefits take effect for NCAA student-athletes Thursday. *NCAA* (2024). Available online at: https://www.ncaa.org/news/2024/8/1/media-center-new-nil-health-and-academic-benefits-take-effect-for-ncaa-student-athletes-aug-1.aspx (Accessed January 3, 2026).

[58] Wogenrich M. Penn State's James Franklin calls NIL ‘the wild wild west.’ *SportsIllustrated.com* (2022). Available online at: https://www.si.com/college/pennstate/football/penn-state-football-james-franklin-nil-wild-wild-west (Accessed January 3, 2026).

[59] Hawkins BJ. The New Plantation: Black Athletes, College Sports, and Predominantly White NCAA Institutions. New York: Palgrave MacMillan (2010).

[60] Murphy D. Donald Trump signs order, seeks to clarify NCAA athletes' employment status. *ESPN* (2025). Available online at: https://www.espn.com/college-sports/story/_/id/45817663/donald-trump-signs-order-seeks-clarify-ncaa-athletes-employment-status?utm_source=chatgpt.com (Accessed December 11, 2025).

[61] White House. Saving college sports. *The White House* (2025). Available online at: https://www.whitehouse.gov/presidential-actions/2025/07/saving-college-sports/ (Accessed December 11, 2025).

[62] Hunnicutt T. Trump signs order aimed at curbing big-money college sports payouts. *Reuters* (2025). Available online at: https://www.reuters.com/sports/trump-signs-order-aimed-curbing-big-money-college-sports-payouts-2025-07-24/ (Accessed December 11, 2025).

[63] On3 NIL. On3 NIL Valuations. *On3* (2025). Available online at: https://www.on3.com/nil/rankings/player/nil-valuations/ (Accessed December 11, 2025).

[64] Brenner R. Dark side of Jacksonian democracy: revolution and racist oppression. *The Activist History Review* (2017). Available online at: https://activisthistory.com/2017/07/14/dark-side-of-jacksonian-democracy-revolution-and-racist-oppression/ (Accessed December 11, 2025).

[65] College Sport Commission. Student athlete deals (2026). Available online at: https://www.collegesportscommission.org/nil (Accessed December 11, 2025).

[66] H.R. 4312 SCORE Act—119th Congress (2025–2026). *Congress.Gov* (2025). Available online at: https://www.congress.gov/bill/119th-congress/house-bill/4312 (Accessed December 11, 2025).

[67] H.R. 1774—119th Congress (2025–2026). *Congress.Gov* (2025). Available online at: https://www.congress.gov/bill/119th-congress/house-bill/1774/text (Accessed December 11, 2025).

[68] Robinson W. ‘I will be the voice for forgotten Americans in this country': Trump says on campaign trail in Pa. Pennlive Patriot-News. (2016). Available online at: https://www.pennlive.com/news/2016/09/i_will_be_the_voice_for_forgot.html (Accessed September 23, 2016).

[69] Abramowitz AI. It's not the Economy, Stupid: The Ideological Foundations of White Working Class Republicanism. The Center for Politics (2025). Available online at: https://centerforpolitics.org/crystalball/its-not-the-economy-stupid-the-ideological-foundations-of-white-working-class-republicanism/ (Accessed April 3, 2025).

[70] Kim J. D.C.’s Black Lives Matter Mural Will be Erased. Look Back at the Iconic Street Painting. NPR. (2025). Available online at: https://www.npr.org/2025/03/08/nx-s1-5321872/dc-black-livesmatter-street-mural-history (Accessed March 8, 2025).

[71] Scalise S. Standardizing NIL for college athletics and protecting student-athletes. Majority Leader. (2025). Available online at: https://www.majorityleader.gov/news/documentsingle.aspx?DocumentID=5754 (Accessed December 3, 2025).

[72] NCSA. NIL (name, Image, and Likeness) Explained. NCSA College Recruiting. (2026). Available online at: https://www.ncsasports.org/name-image-likeness

[73] Magnusen M Todd SY. A fistful of NIL: have we entered a “wild west” recruiting era? J Appl Sport Manag. (2021) 13(2). 10.7290/jasm133hgx

[74] Southall RM Staurowsky EJ. Cheering on the collegiate model: creating, disseminating, and imbedding the NCAA’s redefinition of amateurism. J Sport Soc Issues. (2013) 37(4):403–29. 10.1177/0193723513498606

[75] Wilentz S. The Rise of American Democracy: Jefferson to Lincoln. New York: W. W. Norton (2005).

[76] Keller & Hart v NCAA. NCAA Licensing Litig., No. 10-15387 (9th Cir. 2013). Justia U.S. Law. (2013). Available online at: https://law.justia.com/cases/federal/appellate-courts/ca9/10-15387/10-15387-2013-07-31.html

[77] O'Bannon v. NCAA. No. 14-16601 (9 Cir. 2015). Justia U.S. law. (2015). Available online at: https://law.justia.com/cases/federal/appellate-courts/ca9/14-16601/14-16601-2015-09-30.html

[78] College Athlete Compensation. Congress.gov. (2025). Available online at: https://www.congress.gov/crs-product/LSB11349

